# Barriers of Modern Contraceptive Practices among Asian Women: A Mini Literature Review

**DOI:** 10.5539/gjhs.v5n5p181

**Published:** 2013-07-22

**Authors:** Fatemeh Najafi-Sharjabad, Sharifah Zainiyah Syed Yahya, Hejar Abdul Rahman, Muhamad Hanafiah, Rosliza Abdul Manaf

**Affiliations:** 1 Faculty of Health, Bushehr University of Medical Sciences, Bushehr, Iran; 2 Department of Community Health, Faculty of Medicine and Health Science, Universiti Putra Malaysia, Selangor, Malaysia; 3 Department of Family Medicine, Faculty of Medicine and Health Science, Universiti Putra Malaysia, Selangor, Malaysia

**Keywords:** barriers, factors, use, practice, modern contraceptive methods, Asian women

## Abstract

Family planning has been cited as essential to the achievement of Millennium Development Goals (MDG). Family planning has a direct impact on women's health and consequence of each pregnancy. The use of modern contraception among Asian women is less than global average. In Asia a majority of unintended pregnancies are due to using traditional contraceptive or no methods which lead to induced unsafe abortion. Cultural attitudes, lack of knowledge of methods and reproduction, socio demographic factors, and health service barriers are the main obstacles to modern contraceptive practice among Asian women. Culturally sensitive family planning program, reforming health system, and reproductive health education through mass media to create awareness of the benefits of planned parenthood are effective strategies to improve modern contraceptive practice among Asian women.

## 1. Introduction

Family planning has been cited as essential to the achievement of Millennium Development Goals (MDG) and is an important indicator for tracking progress on improving maternal health (Bernstein & Edouard, 2007). Family planning is one of four pillars with antenatal care, safe delivery, and postnatal care that introduced by the Safe Motherhood Initiative in 1987 to reduce maternal mortality in developing countries, where 99% of all maternal deaths occur (Ahmed et al., 2012).

Family planning allows individuals and couples to anticipate and attain their desired number of children and the spacing and timing of their births. Family planning has a direct impact on women's health and well-being as well as on the consequence of each pregnancy (World Health Organization, 2011^a^). In developing countries about 818 million of sexually active women of reproductive age (15-49) want to avoid pregnancy and delay child bearing for at least two years or want to stop pregnancy and limit their family size. About 140 million of those women (17%) are not using any method of family planning, while 75 million (9%) are using less effective traditional methods. Non contraceptive users and traditional users together (215 million women) are said to have an unmet need for modern contraception (Darroch et al., 2011).

In 2008, use of contraceptive methods prevented over 250 000 maternal deaths through reducing unintended pregnancies. This is equivalent to 40% of the 355 000 maternal deaths for the year. The number of maternal deaths would decrease by a further 30% in developing countries, if all women who wish to avoid pregnancy use an effective contraceptive method (Cleland et al., 2012).

Unmet needs is often described as a problem of access and interpreted as that women do not use contraceptives because they cannot find or afford them. While access is an issue, many other reasons have been cited by women for not using contraceptives, including lack of knowledge, cultural, personal, religious oppositions, health concerns, and fear of side effects. Therefore, just making contraceptives accessible does not guarantee that women will use them (Mills et al., 2010).

In many Asian countries sexuality related topics have greatly remained as a taboo (Adhikari & Tamang, 2009; Agampodi & Agampodi, 2008). Cultural, socioeconomic, and physical norms are identified prominent obstacles of young people for utilizing sexual and reproductive health services (Regmi et al., 2010).

Modern contraceptive methods which include male and female sterilization, intra uterine devices (IUDs), implants, injectables, pills, male and female condoms and spermicides are highly effective in preventing pregnancy, compared with traditional methods, such as withdrawal and periodic abstinence (Singh & Darroch, 2012). Globally, contraceptive use has risen, from 54% in 1990 to 63% in 2007 (World Health Organization, 2011a).

In South Central and Southeast Asia the use of modern contraception is less than global average, with only 47% of married women aged 15–49 years use modern contraceptives, although higher proportions want to prevent pregnancy. The 32% of women, who used a traditional method or no method at all, accounted for 85% of unintended pregnancies in 2008 (United Nations Population Fund, 2009).The rate of annual abortions in Asia slightly increased from 25.9 million to 27.3 million between 2003 and 2008. About 60–65% of abortions in South Central Asia, Southeastern Asia and Western Asia are performed unsafely (Guttmacher Institute, 2012).

In Asia in 2008, 17 000 maternal death which include 12% of all maternal mortality rate were reported due to unsafe abortion (World Health Organization, 2011b). The recent DHS analytical studies show the modern contraceptive prevalence among married women (15-49) in Asian country varied from 14% in Azerbaijan, 20% in Armenia, 22% in Pakistan, 34% in Philippines, 35% in Cambodia, 42% in Jordan, 48% in Bangladesh, 49% in India (Westoff, 2012). The lowest levels of satisfied demand for modern contraception in Asia belong to Armenia and Azerbaijan, at 21% and 27%, respectively. In Cambodia, Pakistan and the Philippines the levels of satisfied demand are below 50% (Westoff, 2012).

Unplanned pregnancies occur when effective contraception is largely inaccessible, or contraceptive method is not used correctly or consistently. It has been estimated that almost 40% unplanned pregnancies occur globally each year as a result of ineffective contraceptive use or failure of method or non-use of contraception (World Health Organization, 2007).

Considering that use of modern contraception is the most reliable way to prevent unintended pregnancy and induced abortion, we carried out a literature search to identify factors that are barriers to modern contraceptive practice among Asian women.

## 2. Method

Six databases were used for the literature search, which include Science Direct, Springer, Wiley-Blackwell, Medline, CINAHL, Google Scholar.

The search terms “barriers” and “factors” were used and combined with “modern contraceptive methods”, “use”, “practice”, and “Asian women”. Over 90 articles published between 2002-2012 were reviewed which included systematic reviews, experimental reports, surveys and qualitative studies. The barriers were categorized in to four main areas: limited knowledge of methods and reproduction, socio demographic factors, cultural, and health service factors

## 3. Results

### 3.1 Limited Knowledge of Methods and Reproduction

Lack of knowledge of modern contraceptive methods and their mechanism of action have been cited one of major reasons for the women's non use of contraception (Khan et al., 2007; Sajid & Malik, 2010; Wu, 2010). Gender disparities in formal schooling have been identified a fundamental structural factor in limiting effective sex education in South Asian and Middle Eastern (UNESCO, 2011). Lacking knowledge of reproductive physiology and fertile period among women and especially adolescent girls may not be effectively assessing their risk of getting pregnant when they have occasional or infrequent sex (Sedgh et al., 2007). A qualitative study among young Vietnamese women revealed they rarely received adequate sex education, which was believed too sensitive a topic to discuss, and out of twelve young women only two in the study had ever used a modern method (Nguyen et al., 2006).

Another qualitative study among Asian immigrant women in Canada explored inadequate knowledge of women's fertile period (Shoveller et al., 2007). Limited knowledge about sexual and reproductive health and poor access to health services forced young people to engage in unsafe sex relationship (Regmi et al., 2010).

In Nepal the women who were exposed to family planning messages through reproductive health staff, were more likely to use modern contraceptives (OR=1.6, p<0.05). The odds of using modern contraceptives methods was higher for women who were exposed to family planning information on radio than unexposed women (OR=1.22, P< 0.01) (Mishra, 2011).

### 3.2 Socio Demographic Factors

As the development progress, socioeconomic development and rapid urbanization lead to a decline in the rate of fertility (Asian Development Bank, 2012). In countries such as Viet Nam, the fertility rate has declined dramatically from 5.4 in 1980 to 1.8 in 2010 (World Bank, 2012). In addition, rich cities such as Shanghai had experienced a drop in birth rate below the population replacement rate. This can be explained due to the occurrence of more opportunities in education and labor force for women in urban areas rather than women from rural areas. Consequently, urban women who are employed tend to have late marriage resulting fewer children. This is further justified by education that women who are working in cities were found to invest most of their time in education (Asian Development Bank, 2012).

Women's education is one of the important factors that influence contraceptive use (Al Riyami et al., 2004; Chavoshi et al., 2004; Saleem & Bobak, 2005). A study in Nepal showed that older women (35 and over), educated, living in urban, working in the business or service sectors were more likely to use modern contraceptive methods (p< 0.05) (Sharma et al., 2011).

In contrast, a survey of South Asian women aged 16 to 50 years, attending inner-city general practices in London, showed that unmarried women (11/13, 85%) were more likely to be using contraception than married women (54/91, [60%]) (OR = 1.4, 99% CI = 1.1 to 1.9). Thirty percent of married women at all ages and 50% (16/32) of women aged more than 30 years who said they had completed their families were not using any contraception (Saxena et al., 2002).

Unmet needs for contraceptive methods are considerably higher among poorer women (Johnson et al., 2008). There is limited political support to provide family planning services for poor people in the Philippines. Since 2004 in Philippine the access of women to supply and family planning services have been reduced. Based on national surveys from 1998 to 2008, the number of women who procured contraceptive methods through pharmacies are increasing. This switch to private sector suppliers reduced access of low-income women and couples to family planning services (Guttmacher Institute, 2010).

The increasing trend of premarital sexual relationship and unintended pregnancies has created a greater need for contraceptives among young women. Sensitivities of sex-related issues in a Muslim-majority country like Malaysia imposed various types of obstacles for young women's access to sexual and reproductive health information, support and practices (Wong, 2012).

In poor countries, young people's economic constraints affect their ability to buy contraceptives or seek sexual and reproductive health services (Chapagain, 2006; Sundby, 2006).

A study among Afghan refugee women in Pakistan showed the use of contraceptive methods among women was higher in subsidized healthcare with increasing age as compared to the women in the non-health subsidy group. For example women aged 25 years in healthcare subsidy group were 0.3 times less likely to use family planning whereas women aged 35 years in the same group were 1.06 times more likely to use it (Raheel et al., 2012).

Ethnic disparities also affect the use of family planning services. Newars ethnicity is the highest among all ethnic groups in Nepal to use contraceptives. Analysis of Nepal Demographic and Health Survey (NDHS), indicated that despite considerable progress in meeting the Millenniumm Development Goals (MDG), different ethnic groups face many barriers to accessing family planning services, because of their illiteracy, poverty, and low social status (Bennett et al., 2008; Mishra, 2011).

### 3.3 Cultural Factors

#### 3.3.1 Religious Factors

The potential influence of different cultures and religions on the acceptance and use of family planning methods by couples have been well documented (Shah et al., 2008; Srikanthan & Reid, 2008).

Analysis of the National Family Health Surveys in India for Muslim and Non-Muslim Differentials in Family Planning showed that Muslim women have greater opposition to family planning. Muslims prefer to use temporary contraceptive methods while the National Family Planning Program promote sterilization. Further, Muslims tend to utilize private-sector services due to greater privacy needs but the program rely on public-sector sources of supply of family planning (Mishra, 2004).

Muslim wives in comparison with non-Muslim wives usually have more children, are more likely to desire additional children, and are less likely to be using contraception when they desire no more children (Morgan et al., 2002).

#### 3.3.2 Fear of Side Effects and Misconceptions

One of the most commonly cited reasons for non-use of contraception is fear of infertility (Williamson et al., 2009; Donati et al., 2000).

Concerns about the side effects, health consequences and inconvenience of methods are particularly high in South and Southeast Asia. Fear of side effects and health concerns have been seen in urban areas of most countries, where barriers related to access seem to be relatively low. Method-related concerns were also common reasons for discontinuation of use among women with unmet need who had used family planning in the past (Sedgh et al., 2007).

Fear of side effects and the belief of being sterile were reported as the major reasons for not using any contraception in Pakistan (Sajid & Malik, 2010).

In a qualitative study among ethnic Korean women living in Canada showed a deep distrust of hormonal contraceptive methods and beliefs that hormonal methods caused permanent harm (Wiebe et al., 2006).

Cambodian women believed modern family planning methods can cause infertility, especially when used before having had at least one child (Vathiny & Hourn, 2009).

Most women mistakenly believed Emergency Contraception (EC) has long-term effects on fertility and causes abortion (Marafie et al., 2007; Shoveller et al., 2007).

#### 3.3.3 Women's Autonomy and Decision Making Power

The link between a woman's level of empowerment and her ability to make decision on reproduction and child bearing has been well documented (Al Riyami et al., 2004; Chavoshi et al., 2004; Saleem & Bobak, 2005).

Due to the multidimensional concept of autonomy, the factors affecting this occurrence were also varied between authors. Most of the literatures in South Asia had reported on women's participation in household decision making, control over financial resources, and mobility (Cleland et al., 1996; Furuta & Salway, 2006). South Asian women are faced with a great disadvantage regarding to autonomy in decision making on their own health care (Senarath & Gunawardena, 2009).

A study among three Asian countries documented non participation of women in household decision making in the majority of Nepal (72.7%) and approximately half of Bangladesh (54.3%) and Indian (48.5%) families. In all the three countries, participation of women in decision making significantly increases with age. Educated women were more likely to participate in decision making than non educated women, OR=1.60; 95% CI = 1.27-2.01 in Nepal, OR=1.71; 95% CI=1.46-1.99 in Bangladesh, and OR=1.67; 95% CI = 1.60-1.74 in India. Urban women were always more likely to be involved in decision making than rural (Senarath & Gunawardena, 2009).

Shah in Pakistan reported that husbands in a majority (67.5 %) of households decide on the desired number of children and contraceptive practice (Shah & Ahman, 2009).

In Cambodia when husbands were the final decision makers about family planning, their wives were less likely than other women to use a contraceptive method (Samandari et al., 2010).

Limitations on women's mobility and prohibition of their accessibility to public places have been documented greatly for South Asian, Middle Eastern, and Central Asian and therefore women could not access to reproductive health services (Al-Riyami et al., 2004; Edmeades et al., 2010; Khan et al., 2007; Pierce & Shaver, 2003).

Researches indicate the limitation on women's mobility in Pakistan and India are connected to their limited access to contraception and abortion services (Edmeades et al., 2010; Khan et al., 2007). Further, a study in Oman revealed women's unmet need for contraception is associated with their decision making power and freedom of movement (Al- Riyami et al., 2004).

There is evidence from Tajikistan that the practice of seclusion or *purdah*, norms against women's presence in public spaces, or other restrictions on women's mobility can pose a direct barrier looking for family planning services (Pierce & Shaver, 2003).

#### 3.3.4 Spouse Approval, Communication and Social Support

Husband's opposition has been reported as the main factor for not using any contraceptive methods among Turkish married women (H. A. Sahin & H. G. Sahin, 2003). The roles of husbands as dominant member in rural areas are important in approving contraceptive practices and family size (Mustafa et al., 2008). Spousal communication about family planning has been proven to increase contraceptive use, even when other factors known to predict contraceptive practice to be controlled (Link, 2011; Wang & Chiou, 2008).

Involving males and obtaining their support and commitment to family planning is crucial for family planning service utilization. Investigation of the influence of spousal communication on the use of family planning methods in rural Nepal and Myanmar showed a strong positive impact of spousal communication on contraceptive use (Link, 2011; Mon & Liabsuetrakul, 2010).

In Cambodia, women who believed that their husbands had a positive attitude towards contraception showed more significantly successful family planning practice (OR=3.4, p<0.001), while women who were nervous about discussing the contraception with their husband were less likely to use the contraceptive method (OR=0.6, p<0.05) (Samandari et al., 2010).

In South Asia, apart from husbands, the role of peers, mothers-in-law, and elders in contraceptive decision making is well documented (Kadir et al., 2003; Kansal et al., 2006; Samandari et al., 2010). Urban women in Pakistan are more likely to use family planning if their mothers-in-law have discussed it with them as an option for their families (Kadir et al., 2003).

Evidence from India showed that involving husbands and mothers-in-law in the intervention increased their support for a longer birth interval and the use of modern contraceptive methods. Moreover, the acceptance of postpartum contraception was significantly increased when the spouse discussed on reproductive issues, such as family planning, the odds ratio was 6.7 to 7.8 times greater among the couple who talked about family planning than when they did not (p<0.01) (Khan et al., 2008).

#### 3.3.5 Preference for a Son

A considerable amount of literature in South Asia, documents that the purpose of using contraception among women is to plan spacing, and number of children. The sex composition of family and the preferences for the sex of future children greatly influence women's decision making about type of contraception practice and when they use it (Jayaraman et al., 2009; Jha et al., 2006; Leone et al., 2003).

The preference for sons in many East and South Asian societies have deep social, economic, and cultural roots. Son preference in India originates from the idea that economic and social benefit of sons is higher compared to daughters (Edmeades et al., 2012; Westley et al., 2007).

In South Asia son preference is higher in urban than in rural areas, in families with more income, and more educated women. On the other hand the picture is more mixed in Central Asia. However son preference is similarly higher in urban than in rural areas, but higher among women with low educational status (Filmer et al., 2008). A study among married women age 15-49 years in Ahmedabad district, India showed the son preference was more in rural areas (94%) than urban areas (81 %; p<0.0001). A majority (93%) of the illiterate women preferred male child whereas 69% of the women who completed graduation had the preference for son (p<0.01) (Chavada & Bhagyalaxmi, 2009).

#### 3.3.6 To Prove Fertility Soon after Marriage

Women and men face strong social pressure to prove their fertility as soon as possible after marriage (McCleary-Sills et al., 2012). Marriage structures in many countries especially in Asia have been set up to maximize fertility and also to ensure early childbearing.

While this pattern has shifted significantly to later marriage and childbearing in East Asian countries, it is still common in South Asia. Countries such as India, Nepal, Yemen, have significant high rates of early marriage and early childbearing (Malhotra et al., 2011; Mathur et al., 2003; McIntyre, 2006).

Strong social norms against delaying a first birth after marriage in countries like India with a high rates of adolescent childbearing make it difficult to eliminate this misconception (Rahman et al., 2010). Reflecting similar norms among ever married women in Jordan, only 12% approved of contraceptive use before the first birth, while the family planning program strongly support contraceptive practice (Storey, 2008).

#### 3.3.7 Social Stigma and Embarrassment

Embarrassment and poor negotiation skills impose barriers to access sexual health information and services (Roberts et al., 2005; Van Teijlingen et al., 2007). Rural young people are more likely to be embarrassed than urbanites because there is a concern of stigmatization from local people in the rural areas (Regmi et al., 2010).

Studies in Nepal and India have found that adolescents are reluctant to go to clinics and pharmacies to obtain contraceptives. They fear to be recognized by providers or people in their community and would negatively label them as sexually active (Mathur et al., 2004; Pande, 2006). Research among Asian immigrant women had documented that they were not comfortable requesting EC from a health provider of the same ethnicity. This group of women assume that this act may result in chastise or gossip about them.

They also feel uncomfortable to request EC from male doctors or pharmacists (Shoveller et al., 2007).

### 3.4 Health Service Factors

Supply of methods and services is one of the most common cited reasons by married women for not using contraception (Sedgh et al., 2007).

Non-availability of contraceptive, cost, long waiting hours at the center, shortage of the female staff and cost were reported as unsatisfactory variables in Pakistan (Shah et al., 2008).

More than 80% of doctors across six cities in India refused women’s’ access to sterilization if they were unmarried, had low parity, young, or lacking consent of the spouse (Nanda et al., 2011). Studies on women's reproductive health have revealed that many health providers did not support the use of EC in Islamic countries because of concerns about promoting promiscuity (Mir & Malik, 2010; Sevil et al., 2006). In Indonesia medical practitioners prescribe EC pills infrequently. Most of obstetricians and gynecologist do not support EC be available Over the Counter without prescription (Dyna & Lisa, 2005). A cross sectional study among Turkish health providers indicated a few of them included EC in routine consultations. Half of the health providers thought disseminating information about EC would encourage young people to have unprotected sexual intercourse. Majority worried that increasing awareness of this method would lead to raising sexually transmitted infections because people would stop using barrier methods (Sevil et al., 2006).

## 4. Conclusion and Recommendation

Review of literatures provide evidences that most obstacles to modern contraceptive use in Asian countries are related to limited knowledge, women's autonomy, misconception, cultural and health system barriers.

The studies revealed most of women particularly adolescents lacked knowledge of reproductive organ physiology and contraceptive methods. Disseminating information on sexual health through mass media would be a possible means to urban youths. Also sexual health education by trained peer educators can be an effective method of improving the knowledge of young people on the issues of sexual and reproductive health (Price & Knibbs, 2009).

There is a need to introduce reproductive and sex education in schools to prepare the young for healthy and responsible living. Reproductive health programme designers should focus on developing negotiation skills in young people. Establishment of youth-friendly service centers in convenient places and providing essential materials would encourage young people to use sexual health services (Regmi et al., 2010).

Evidence indicated that respecting young people's confidentiality is an effective way of preventing teenage pregnancy (Swann et al., 2003). Mass media communication campaigns can raise awareness of the benefits of family planning and of responsible parenthood. Family planning programs should also reach out to broader audiences, such as religious and community leaders (Roudi-Fahimi et al., 2012).

The autonomy of women was found to be a significant factor that influences contraceptive use in Asian population. Studies have suggested that greater gender equality may encourage women's autonomy and may facilitate the uptake of contraception because of increased female participation in decision making (Hakim et al., 2003).

Women's autonomy in decision making on health care should be improved in Asia particularly in South Asian region, and this could be achieved by promoting higher education and gainful employment for women. Women with higher education are more likely able to resist subjugation and to attain greater power in decision-making (Mustafa et al., 2008; Senarath & Gunawardena, 2009).

One effective strategy for improving family planning practice and reducing unmet need is reforming service delivery systems and well implemented interventions. Studies have shown that redesigning health system improve family planning behaviors even in areas with widespread poverty, low literacy, and largely rural populations (Nyonator et al., 2007; Phillips et al., 2007).

Coordination or integration of services as complementary way to reach women and reduce missed opportunities to provide family planning services. For example in a study in Turkey providers interviewed clients about their need for family planning after offering routine services, such as children's vaccinations and checkups. They found that 43% of clients had an unmet need for a modern contraceptive method. Referrals to the family planning unit led about two fifths of them to adopt a method that same day (Cali et al., 2004). Improving the quality of family planning services not only attract new clients but can also help prevent contraceptive discontinuation (Ramchandran, 2007). Modern family planning services include counseling, provision of contraceptives and follow-up.

Providers should be trained to give women correct information on contraceptive methods, especially on side effects and how to manage them. The women who are in postpartum period, breastfeeding, or approaching menopause the likelihood of becoming pregnant need to be advised by health providers.

These women also should be counseled on choosing appropriate contraceptive methods (Roudi-Fahimi et al., 2012).

The collaboration between private and public health sectors are needed to ensure that family planning supplies and services are available and accessible to those who need them.

Reproductive health programmer may pay special attention to those with unmet need for contraception to prevent unwanted or mistimed pregnancies, the marginalized groups who may lack knowledge and access to family planning, and users of traditional methods.

In order to promote modern contraception among Asian women the following are suggested.

Public education programmes through mass media (national TV, radio, newspapers, magazines, internet, public forums, and interfaith dialogues) can raise the awareness of people regarding family planning and its benefits.

Re- educating the health providers like continuous medical education programmes for GPs, medical officers and paramedics.

Also, special family planning clinic for high risk cases involving husbands and youths should be conducted to increase the awareness of family planning.

Addressing the main socio cultural concerns is an important strategy to improve acceptance of family planning. Family planning programs should be sensitive to culture and traditional perspectives to birth control. An awareness of cultural and religious beliefs helps health care providers give accurate information that shows a woman's beliefs and concerns (Roberts, 2008).

Male participation in sharing the responsibility to practice and support family planning is identified as a vital strategy in increasing the contraceptive prevalence rate. In Asian countries reproductive health services are more focused on women and family planning services and information are not targeted towards men (Abdul Manaf & Manaf, 2010; Tey, 2010). Therefore, it is important to neutralize the stereotyping or, “feminization” of the service as a whole (Abdul Manaf & Manaf, 2010). Involving men in family planning program need comprehensive multilevel approach from policy to infrastructure and service delivery (Abdul Manaf & Manaf, 2010). Also, reproductive health educational program need to engage men to gain further support and to encourage husbands to share more responsibility in family planning. Implementing these strategies among Asian countries may empower women to use effective modern contraceptive methods and improving maternal health which can helps governments to achieve their development goals.

## References

[ref1] Abdul Manaf R, Manaf M (2010). Male participation and sharing of responsibility in strengthening family planning activities in Malaysia. Malaysian Journal of Public Health Medicine.

[ref2] Adhikari R, Tamang J (2009). Premarital sexual behavior among male college students of Kathmandu, Nepal. BMC Public Health.

[ref3] Agampodi S. B, Agampodi T. C (2008). Adolescents perception of reproductive health care services in Sri Lanka. BMC health services research.

[ref4] Ahmed S, Li Q, Liu L, Tsui A. O (2012). Maternal deaths averted by contraceptive use: an analysis of 172 countries. The Lancet.

[ref5] Al Riyami A, Afifi M, Mabry R. M (2004). Women's autonomy, education and employment in Oman and their influence on contraceptive use. Reproductive health matters.

[ref6] Asian Development Bank (2012). Key Indicators for Asia and the Pacific 2012: Green Urbanization in Asia, special chapter.

[ref7] Bennett L, Dahal D. R, Govindasamy P, Measure D (2008). Caste, Ethnic, and Regional Identity in Nepal: Further Analysis of the 2006 Nepal Demographic and Health Survey: Population Division, Ministry of Health and Population, Government of Nepal.

[ref8] Bernstein S, Edouard L (2007). Targeting access to reproductive health: giving contraception more prominence and using indicators to monitor progress. Reproductive health matters.

[ref9] Cali S, Kalaca S, Sarikaya O (2004). Minimizing missed opportunities: an approach to decrease the unmet need for family planning. European J. of Contraception and Reproductive Healthcare.

[ref10] Chapagain M (2006). Conjugal Power Relations and Couples’ Participation in Reproductive Health Decision-Making Exploring the Links in Nepal. Gender, Technology and Development.

[ref11] Chavada M, Bhagyalaxmi A (2009). Effect of socio-cultural factors on the preference for the sex of children by women in ahmedabad district. Health and Population: Perspectives and Issues.

[ref12] Chavoshi M. H, Abbasi-Shavazi M. J, McDonald P (2004). Women's autonomy and reproductive behavior in Iran. Paper presented at the 12th Biennial Conference of the Australian Population Conference, Canberra.

[ref13] Cleland J, Conde-Agudelo A, Peterson H, Ross J, Tsui A (2012). Contraception and health. The Lancet.

[ref14] Cleland J, Kamal N, Sloggett A, Links between fertility regulation and the schooling and autonomy of women in Bangladesh (1996). Girls’ Schooling, Women’ Autonomy and Fertility Change in South Asia.

[ref15] Darroch J. E, Sedgh G, Ball H (2011). Contraceptive Technologies: Responding to women's needs.

[ref16] Donati S, Hamam R, Medda E (2000). Family planning KAP survey in Gaza. Social science & medicine.

[ref17] Edmeades J, Lee Rife S. M, Malhotra A (2010). Women and Reproductive Control: The Nexus between Abortion and Contraceptive Use in Madhya Pradesh, India. Studies in Family Planning.

[ref18] Edmeades J, Pande R. P, Falle T, Krishnan S (2012). Son preference and sterilisation use among young married women in two slums in Bengaluru city, India. Global public health.

[ref19] Filmer D, Friedman J, Schady N (2008). Development, modernization, and son preference in fertility decisions. World Bank Policy Research Working Paper.

[ref20] Furuta M, Salway S (2006). Women's position within the household as a determinant of maternal health care use in Nepal. International family planning perspectives.

[ref21] Glanz K, Rimer B. K, Viswanath K (2008). Health behavior and health education: theory, research, and practice: Jossey-Bass.

[ref22] Guttmacher Institute (2010). Facts on Barriers to Contraceptive Use in the Philippines.

[ref23] Guttmacher Institute (2012). Facts on Abortion in Asia.

[ref24] Hakim A, Salway S, Mumtaz Z (2003). Women's autonomy and uptake of contraception in Pakistan. Asia Pacific Population Journal.

[ref25] Jayaraman A, Mishra V, Arnold F (2009). The relationship of family size and composition to fertility desires, contraceptive adoption and method choice in South Asia. International Perspectives on Sexual and Reproductive Health.

[ref26] Jha P, Kumar R, Vasa P, Dhingra N, Thiruchelvam D, Moineddin R (2006). Low male-to-female sex ratio of children born in India: national survey of 1Â·1 million households. The Lancet.

[ref27] Jha S. M, Chaurasia R, Jha B (2010). Knowledge about condoms among adolescents in Kathmandu Valley. Journal of Nepal Paediatric Society.

[ref28] Johnson K, Bradley S. E. K, MEASURE D (2008). Trends in Economic Differentials in Population and Health Outcomes: Further Analysis of the 2006 Nepal Demographic and Health Survey: Population Division, Ministry of Health and Population, Government of Nepal.

[ref29] Kadir M. M, Fikree F. F, Khan A, Sajan F (2003). Do mothers-in-law matter? Family dynamics and fertility decision-making in urban squatter settlements of Karachi, Pakistan. Journal of biosocial science.

[ref30] Kansal A, Kandpal S, Mishra P (2006). Reasons for not practicing contraception in a rural population of Dehradun District. Journal of Communicable Diseases.

[ref31] Khan M. E, Sebastian M. P, Sharma U, Idnani R, Kumari K, Maheshwari B, Ashraf S, Promoting healthy timing and spacing of births in India through a community-based approach (2008). Population Council.

[ref32] Khan M. H, Humayun Shah S, Saba N, Anwar S, Ahmad I, Babar K. S, Gul B (2007). Study of contraceptive user women in Di Khan, Pakistan. Biomedica.

[ref33] Leone T, Matthews Z, Zuanna G. D (2003). Impact and determinants of sex preference in Nepal. International family planning perspectives.

[ref34] Link C. F (2011). Spousal Communication and Contraceptive Use in Rural Nepal: An Event History Analysis. Studies in Family Planning.

[ref35] Malhotra A, Warner A, McGonagle A, Lee-Rife S (2011). Solutions to End Child Marriage What the Evidence Shows. International Center for Research on Women.

[ref36] Marafie N, Ball D. E, Abahussain E (2007). Awareness of hormonal emergency contraception among married women in a Kuwaiti family social network. European Journal of Obstetrics & Gynecology and Reproductive Biology.

[ref37] Mathur S, Greene M, Malhotra A (2003). Too young to wed: the lives, rights and health of young married girls: International Center for Research on Women.

[ref38] Mathur S, Mehta M, Malhotra A (2004). Youth reproductive health in Nepal: Is participation the answer?: International Center for Research on Women.

[ref39] McCleary-Sills J, McGonagle A, Malhotra A (2012). Women's demand for reproductive control. Understanding and addressing gender barriers. International Center for Research on Women.

[ref40] McIntyre P (2006). Married adolescents: no place of safety.

[ref41] Mills S, Bos E, Suzuki E (2010). Unmet need for contraception. Human Development Network, World Bank.

[ref42] Mir A. S, Malik R (2010). Emergency contraceptive pills: Exploring the knowledge and attitudes of community health workers in a developing Muslim country. North American Journal of Medical Sciences.

[ref43] Mishra M (2011). Ethnic Disparities in Contraceptive Use and its Impact on Family Planning Program in Nepal. Nepal Journal of Obstetrics and Gynaecology.

[ref44] Mishra V. K (2004). Muslim/non-muslim differentials in fertility and family planning in India.

[ref45] Mon M. M, Liabsuetrakul T (2010). Predictors of Contraceptive Use among Married Youths and Their Husbands in a Rural Area of Myanmar. Asia-Pacific Journal of Public Health.

[ref46] Morgan S. P, Stash S, Smith H. L, Mason K. O (2002). Muslim and non Muslim differences in female autonomy and fertility: evidence from four Asian countries. Population and Development Review.

[ref47] Mustafa R, Afreen U, Hashmi H. A (2008). Contraceptive knowledge, attitude and practice among rural women. J Coll Physicians Surg Pak.

[ref48] Nanda P, Achyut P, Mishra A, Calhoun L (2011). Measurement, Learning & Evaluation of the Urban Health Initiative: Uttar Pradesh, India, Baseline Survey 2010.

[ref49] Nguyen H. N, Liamputtong P, Murphy G (2006). Knowledge of contraceptives and sexually transmitted diseases and contraceptive practices amongst young people in Ho Chi Minh City, Vietnam. Health care for women international.

[ref50] Nyonator F. K, Akosa A. B, Awoonor-Williams J. K, Phillips J. F, Jones T. C (2007). Scaling up experimental project success with the Community-based Health Planning and Services initiative in Ghana. Scaling up Health Service Delivery: From Pilot Innovations to Policies and Programmes.

[ref51] Pande R (2006). Improving the Reproductive Health of Married and Unmarried Youth in India: Evidence of Effectiveness and Costs from Community-based Interventions, Final Report of the Adolescent Reproductive Health Program in India: ICRW.

[ref52] Phillips J. F, Nyonator F. K, Jones T. C, Ravikumar S (2007). Evidence-based scaling up of health and family planning service innovations in Bangladesh and Ghana. Scaling up Health Service Delivery: From Pilot Innovations to Policies and Programmes.

[ref53] Pierce E, Shaver T (2003). CARE International in Tajikistan: Final Evaluation Report-Varzob Reproductive Health Improvement Project. USAID & CARE.

[ref54] Price N, Knibbs S (2009). How Effective is Peer Education in Addressing Young People's Sexual and Reproductive Health Needs in Developing Countries?. Children & Society.

[ref55] Raheel H, Karim M. S, Saleem S, Bharwani S (2012). Knowledge, Attitudes and Practices of Contraception among Afghan Refugee Women in Pakistan: A Cross-Sectional Study. Plos one.

[ref56] Rahman M, Daniel E (2010). A Reproductive Health Communication Model That Helps Improve Young Women's Reproductive Life and Reduce Population Growth: The Case of PRACHAR from Bihar, India. Pathfinder.

[ref57] Ramchandran D (2007). Developing a continuing-client strategy. Population reports. Series J: Family planning programs.

[ref58] Regmi P. R, van Teijlingen E, Simkhada P, Acharya D. R (2010). Barriers to sexual health services for young people in Nepal. Journal of health, population, and nutrition.

[ref59] Roberts A. B, Oyun C, Batnasan E, Laing L (2005). Exploring the social and cultural context of sexual health for young people in Mongolia: implications for health promotion. Social science & medicine.

[ref60] Roberts E (2008). Breaking the Contraceptive Barrier: Techniques for Effective Contraceptive Consultations. Association of Reproductive Health Professionals.

[ref61] Roudi-Fahimi F, Monem A. A, Ashford L, El-Adawy M (2012). Women's need for family planning in Arab countries. United Nations Population Fund. Population Reference Bureau. July 2012.

[ref62] Sahin H. A, Sahin H. G (2003). Reasons for not using family planning methods in Eastern Turkey. European J. of Contraception and Reproductive Healthcare.

[ref63] Sajid A, Malik S (2010). Knowledge, Attitude and Practice of Contraception Among Multiparous Women at Lady Aitchison Hospital, Lahore. Annals of King Edward Medical University.

[ref64] Saleem S, Bobak M (2005). Women's autonomy, education and contraception use in Pakistan: a national study. Reprod Health.

[ref65] Samandari G, Speizer I. S, O’ Connell K (2010). The role of social support and parity on contraceptive use in Cambodia. International Perspectives on Sexual and Reproductive Health.

[ref66] Saxena S, Oakeshott P, Hilton S (2002). Contraceptive use among South Asian women attending general practices in southwest London. The British Journal of General Practice.

[ref67] Sedgh G, Hussain R, Bankole A, Singh S (2007). Women with an unmet need for contraception in developing countries and their reasons for not using a method: Alan Guttmacher Institute.

[ref68] Senarath U, Gunawardena N. S (2009). Women's autonomy in decision making for health care in south Asia. Asia-Pacific Journal of Public Health.

[ref69] Sevil U, Yanikkerem E, Hatipoglu S (2006). A survey of knowledge, attitudes and practices relating to ECamong health workers in Manisa, Turkey. Midwifery.

[ref70] Shah I, Ahman E (2009). Unsafe abortion: global and regional incidence, trends, consequences, and challenges. J Obstet Gynaecol Can.

[ref71] Shah N. A, Nisar N, Qadri M. H (2008). Awareness and pattern of utilizing family planning services among women attending Urban Health Care Center Azizabad Sukkur. Pak J Med Sci July-September.

[ref72] Sharma S. K, Ghimire D. R, Pratap N (2011). Ethnic differentials of the impact of Family Planning Program on contraceptive use in Nepal. Demographic Research.

[ref73] Shoveller J, Chabot C, Soon J. A, Levine M (2007). Identifying barriers to ECuse among young women from various sociocultural groups in British Columbia, Canada. Perspectives on Sexual and Reproductive Health.

[ref74] Singh S, Darroch J. E (2012). Adding it up: costs and benefits of contraceptive services. Estimates for 2012.

[ref75] Srikanthan A, Reid R. L (2008). Religious and cultural influences on contraception. JOGC-TORONTO.

[ref76] Storey D (2008). Communication Partnership for Family Health Midterm Survey Main Report. Johns Hopkins University Bloomberg School of Public Health Center for Communications Programs, Baltimore, MD and the Jordan health Communication Partnership, Amman, Jordan.

[ref77] Sundby J (2006). Young people's sexual and reproductive health rights. Best Practice & Research Clinical Obstetrics & Gynaecology.

[ref78] Swann C, Bowe K, Kosmin M, McCormick G (2003). Teenage pregnancy and parenthood: a review of reviews. Evidence briefing: Health Development Agency London.

[ref79] Syahlul D. E, Amir L. H (2005). Do Indonesian medical practitioners approve the availability of EC over-the-counter? A survey of general practitioners and obstetricians in Jakarta. BMC women's health.

[ref80] Tey N. P, Hosseini H, Mie N. Y (2010). The role of men in contraceptive use in Indonesia. New Delhi, India, November 2010.

[ref81] UNESCO (2011). Global Education Digest: Comparing Education Statistics across the World. Montreal, Canada: The UNESCO Institute for Statistics.

[ref82] United Nations Population Fund (UNFPA) (2009). South Central and Southeast Asia Facts on Investing in Family Planning and Maternal and Newborn Health. UNFPA, Guttmacher Institute.

[ref83] Van Teijlingen E, Reid J, Shucksmith J, Harris F, Philip K, Imamura M, Penney G (2007). Embarrassment as a key emotion in young people talking about sexual health. Sociological research online.

[ref84] Vathiny O. V, Hourn K. K (2009). Barriers to Contraceptive Use in Cambodia. Kuala Lumpur.

[ref85] Wang R. H, Chiou C. J (2008). Relative contribution of intrapersonal and partner factors to contraceptive behavior among Taiwanese female adolescents. Journal of Nursing Scholarship.

[ref86] Westley S. B, Choe M. K, Center E. W (2007). How does son preference affect populations in Asia?: East-West Center.

[ref87] Westoff C. F (2012). Unmet Need for Modern Contraceptive Methods. DHS Analytical Studies No. 28.

[ref88] Wiebe E. R, Henderson A, Choi J, Trouton K (2006). Ethnic Korean women's perceptions about birth control. Contraception.

[ref89] Williamson L. M, Parkes A, Wight D, Petticrew M, Hart G. J (2009). Limits to modern contraceptive use among young women in developing countries: a systematic review of qualitative research. Reproductive Health.

[ref90] Wong L. P (2012). An exploration of knowledge, attitudes and behaviours of young multiethnic Muslim-majority society in Malaysia in relation to reproductive and premarital sexual practices. BMC Public Health.

[ref91] World Bank (2012). World Development Indicators Online.

[ref92] World Health Organization (2007). Unsafe abortion. Global and regional estimates of the incidence of unsafe abortion and associated mortality in 2003 (Fifth edition). Geneva.

[ref93] World Health Organization (2011a). Family Planning.

[ref94] World Health Organization (2011b). Unsafe abortion: global and regional estimates of the incidence of unsafe abortion and associated mortality in 2008.

[ref95] Wu L (2010). A Survey on the Knowledge, Attitude, and Behavior Regarding Contraception Use among Pregnant Teenagers in Beijing, China. Clinical Nursing Research.

